# When complexity science meets implementation science: a theoretical and empirical analysis of systems change

**DOI:** 10.1186/s12916-018-1057-z

**Published:** 2018-04-30

**Authors:** Jeffrey Braithwaite, Kate Churruca, Janet C. Long, Louise A. Ellis, Jessica Herkes

**Affiliations:** Centre for Healthcare Resilience and Implementation Science, Australian Institute of Health Innovation, Macquarie University, Level 6, 75 Talavera Road, North Ryde, NSW 2109 Australia

**Keywords:** Complexity science, Implementation science, Translation, Improvement, Change, Systems innovation, Health and medical research, Take up, Speed, Culture

## Abstract

**Background:**

Implementation science has a core aim – to get evidence into practice. Early in the evidence-based medicine movement, this task was construed in linear terms, wherein the knowledge pipeline moved from evidence created in the laboratory through to clinical trials and, finally, via new tests, drugs, equipment, or procedures, into clinical practice. We now know that this straight-line thinking was naïve at best, and little more than an idealization, with multiple fractures appearing in the pipeline.

**Discussion:**

The knowledge pipeline derives from a mechanistic and linear approach to science, which, while delivering huge advances in medicine over the last two centuries, is limited in its application to complex social systems such as healthcare. Instead, complexity science, a theoretical approach to understanding interconnections among agents and how they give rise to emergent, dynamic, systems-level behaviors, represents an increasingly useful conceptual framework for change. Herein, we discuss what implementation science can learn from complexity science, and tease out some of the properties of healthcare systems that enable or constrain the goals we have for better, more effective, more evidence-based care. Two Australian examples, one largely top-down, predicated on applying new standards across the country, and the other largely bottom-up, adopting medical emergency teams in over 200 hospitals, provide empirical support for a complexity-informed approach to implementation. The key lessons are that change can be stimulated in many ways, but a triggering mechanism is needed, such as legislation or widespread stakeholder agreement; that feedback loops are crucial to continue change momentum; that extended sweeps of time are involved, typically much longer than believed at the outset; and that taking a systems-informed, complexity approach, having regard for existing networks and socio-technical characteristics, is beneficial.

**Conclusion:**

Construing healthcare as a complex adaptive system implies that getting evidence into routine practice through a step-by-step model is not feasible. Complexity science forces us to consider the dynamic properties of systems and the varying characteristics that are deeply enmeshed in social practices, whilst indicating that multiple forces, variables, and influences must be factored into any change process, and that unpredictability and uncertainty are normal properties of multi-part, intricate systems.

“*As complex as things are today, everything will be more complex tomorrow.*”

— K. Kelly in *Out of Control: The New Biology of Machines, Social Systems and the Economic World* [1]

“*One question … is whether the implementation of radical organizational change in health care is actually the core issue … there are many small-scale improvements and experimental projects … thus the primary issue is one of evaluation and spread.*”

— L. Fitzgerald in *Challenging Perspectives on Organizational Change in Health Care* edited by L. Fitzgerald and A. M. McDermott [2]

## Background

In what now seems to us like the distant past, yet, in reality, was merely a decade or so ago, medical scientists believed that the translation of research evidence into practice followed a prescribed set of research steps, moving from test tube to needle, or bench to bedside. It was common to apply the concept of a ‘pipeline’ as a heuristic for understanding research uptake. Adherents to this view frequently diagrammed the process as a linear one, conceptualizing interventions through a series of stages starting from the laboratory, into the randomized trial environment, and then across real-world settings.

**Fig. 1:**
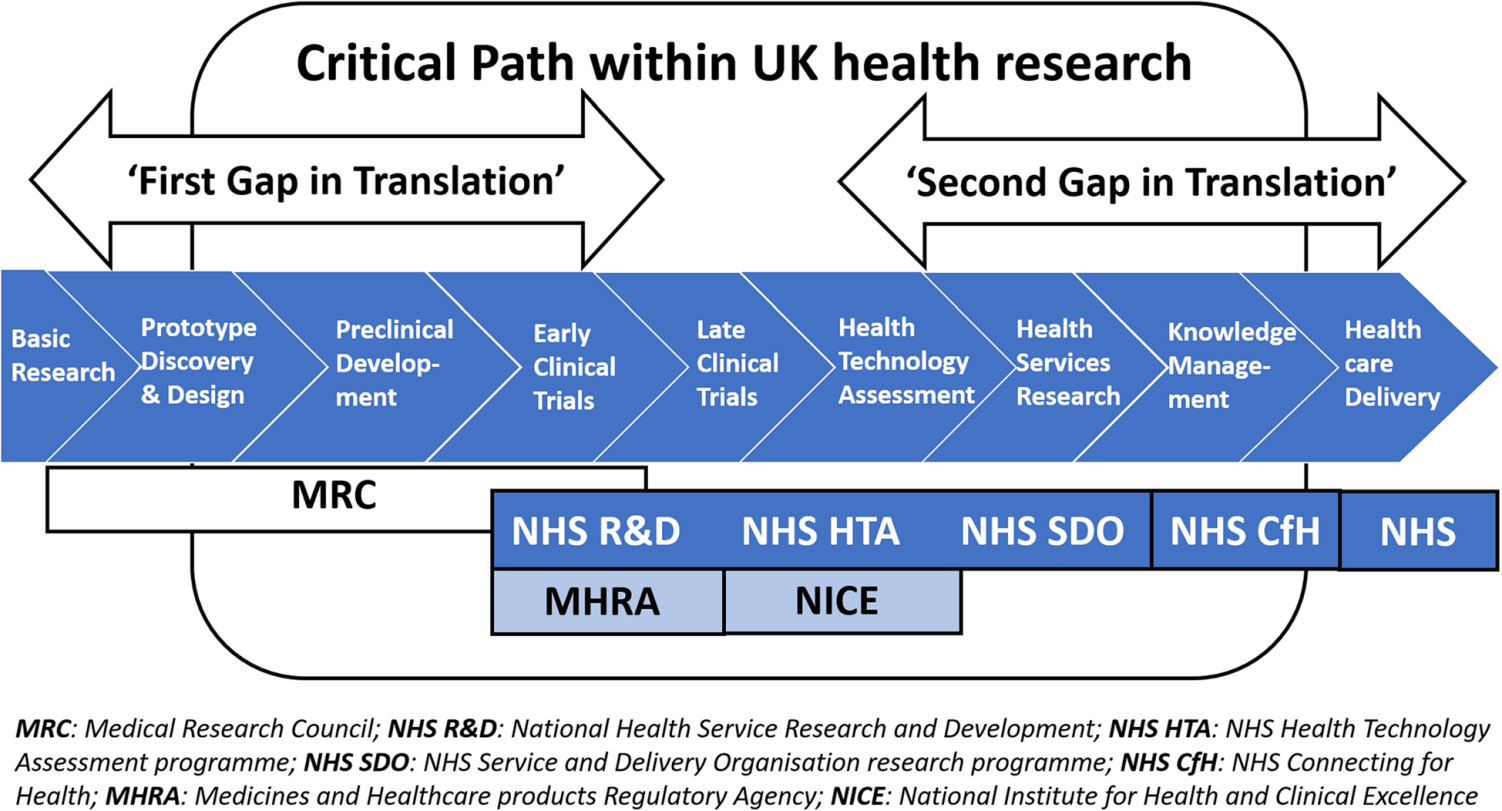
Example of a causal linear approach for the translation of health research into practice. Source: Cooksey [3]. Use of this image is supported by an Open Government License (http://nationalarchives.gov.uk/doc/open-government-licence/version/3/)

Such models implicitly assumed that those on the clinical frontlines would naturally provide new types of care, such as novel pharmaceuticals, practices, or innovative technologies, based on the latest evidence, and all heavily informed by upstream research. While various research pipeline models were proposed over the years, all were similar in that research evidence was assumed to advance in a rational, step-wise manner. One influential model, described in the Cooksey report [3] (Fig. 1), was developed following a review of health research funding in the UK examining the critical pathways to successful research translation; it is often referred to, and equivalent models have been developed in other countries [4, 5].

However, the linear, rational way in which such a model assumes that evidence is converted into practice masks the complexity of the research–practice ecosystem [6, 7]. It hides much of what is important in trying to accomplish evidence-based medicine, namely, that basic research is fundamentally risky and often does not produce any useable breakthrough; that some ideas never even reach the prototype stage, let alone pre-clinical development; that even if developments progress to a trial, this may prove unsuccessful; that health services research is relatively poorly funded and thus implementers often fall short of truly understanding how socio-professional systems work in practice; and that the ‘translation gaps’ (more like chasms) between research findings and their use in practice often cannot be bridged [8–10].

This traditional manner of thinking about research pathways was founded on a Newtonian-style, clockwork universe paradigm, representing a mechanistic and reductionist view of the way the world works, dominated by the randomized clinical trial and precision measurement. In reality, when we deal with non-mechanical human systems, this view has serious limitations [11]. To extend the metaphor, in contrast to a Newtonian view, the health system is more quantum mechanical than classically clockwork, and is characterized by uncertainty, emergence, and embedded unpredictability. Participants exert effects on the system; sometimes, the system appears wave-like (akin to group behaviors), sometimes particle-like (with individual agents’ efforts having influence), and it changes once measured or observed, because measurers and observers are entangled within the system and each other. The health system is probabilistic and stochastic rather than deterministic and causal.

## Shifting the paradigm

Some 10 to 15 years ago, several thinkers began to realize the limitations inherent in the pipeline idea [12] as it became increasingly obvious that getting evidence into practice was much harder than earlier proponents believed. This recognition came from the knowledge and understanding of human systems that had been accumulating in sociology, ecology, and evolutionary biology ever since the 1940s, and with antecedents even earlier, which we can loosely call ‘systems thinking’.

The systems view is based on several fundamental ideas, essentially, that all systems are composed of a set of seemingly discrete but actually interdependent components, defined not just by their inter-relations but by the permeable and shifting boundaries between them. The components (people, technology, artefacts, equipment) are combined haphazardly and in unexpected ways, aggregating to be more than the sum of their parts, and are characterized by eddying, recurring patterns of behavior. Key moments in the path of articulating a systems view of the world arose through the work of many theorists, but management scientist Peter Checkland [13], biologist Ludwig von Bertalanffy [14], and organizational theorist Andrew Van de Ven [15] can be used as proxy exemplars.

Checkland’s pioneering work [13], beginning in the 1960s, was encapsulated under the title ‘soft systems methodology’. This approach differentiated between hard systems, represented by relatively rigid techniques, technology, artefacts, and equipment, and soft systems, which involve the learning that occurs in fuzzy, ill-defined circumstances as people navigate across time in messy ecosystems.

Von Bertalanffy’s ideas date decades earlier, and his development of ‘General System Theory’ laid the platform for much of the later work. He, in turn, drew on even earlier sociological, mathematical, and biological research and theories, and by approximately 1946 he had assembled General System Theory, applying universal principles and espousing the ontological underpinnings for the interactive and dynamic nature of social organization and structuring [14, 16].

Andrew Van de Ven’s work built on this systems approach through the 1990s, culminating in his book *The Innovation Journey* [15], which proved timely and useful for those interested in translational research processes. An organizational theorist, he too distinguished between linear conceptualizations and more unpredictable, iterative approaches, but made a further distinction between the two world views. When speaking about innovation, he argued that attention must be paid to fluidity, messiness, and even chaotic tendencies. Van de Ven noted, through a series of case studies, that innovation often manifests not progressively in a step-by-step manner, but recursively, and always diverging from aspired-to pathways. He encapsulated this duality by showing the implicit mechanistic assumptions made in the literature, in stark contrast with what hke actually saw when he researched and observed innovative practices (Table 1).

**Table 1:** Assumptions and observations about core innovation concepts

Concept	Linear causal thinking	Systems thinking
Ideas	One invention, operationalized	Reinvention, proliferation, reimplementation, discarding, and termination
Innovator(s)	An entrepreneur with a fixed set of full-time people over time	Many entrepreneurs and other players, sometimes on-track and sometimes distracted, fluidly engaging and disengaging over time in a variety of roles
Transaction	A defined network of people or firms working out details of an idea between themselves	Expanding, contracting, and flexing networks of partisan stakeholders who converge and diverge on ideas
Context	The environment provides opportunities and constraints on the innovation process	The innovation process is captured by political and cultural features, and creates opponents or is constrained by multiple enacted environments
Process	Simple, orderly, cumulative sequences of stages or phases	Multiple messy, imprecise journeys; many divergent, parallel and convergent paths; some related, others not
Outcomes	Final result predictable; a stable new order comes into being	Final result indeterminate; many in-process perturbations, assessments and spinoffs; integration of any new order with old orders

Source: Modified from Van de Ven et al. [15]

For Van de Ven and his intellectual successors, the trajectories to an innovative outcome always have several variations, multiple pathways, unanticipated processes and results, and exhibit conflict between stakeholders. People flex and adjust, accommodating to local conditions, and always deviate from idealized pathways.

Innovation processes for Van de Ven are neither stable and predictable nor stochastic and chaotic. Being an innovator implies working with inherent unpredictability, sometimes with random effects, and dealing with the multiplicity of internal and external forces that impinge on and are intrinsic to the journey. Sometimes, innovators need to run with the pack, and at other timess do so in opposition. Persistence in the face of setbacks and an ability to work with, or simply just understand, multiple agents who inhabit indistinct, orthogonal, or oppositional cultures and subcultures, facing sometimes destructive and sometimes constructive politics, and experiencing periods of inactivity, are all features of the innovation journey.

## Bringing the systems view together

**Fig. 2:**
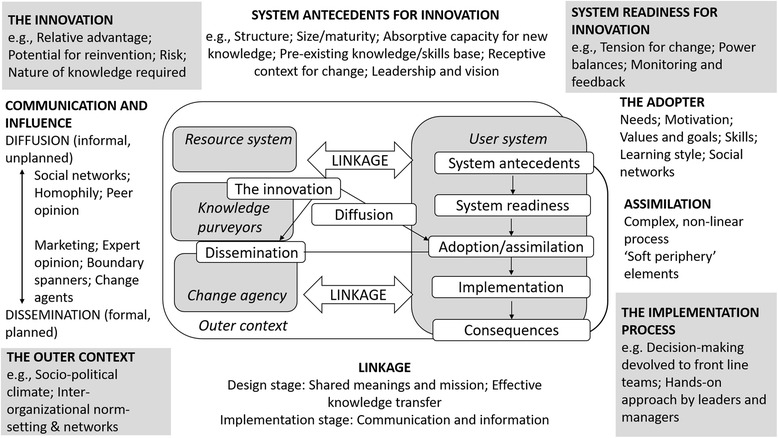
Conceptual model – determinants of diffusion, dissemination, and implementation of innovations in health services. Source: Modified from Greenhalgh et al. [12]. Written permission granted by Wiley Global Permissions

From 2004, this rich theorizing and new-fashioned conceptualizing of the ontology of improvement pathways began to be applied more concertedly to healthcare. Many of these ideas converged in Greenhalgh’s work on the diffusion of innovation, where she and her colleagues brought together disparate research in an influential paper that provided an extended systems model articulating the intricacies, problematics, and minutiae of getting evidence into practice (Fig. 2) [12]. The Greenhalgh model suggested four pivotal systems factors important for innovation, namely the innovation itself, and its characteristics; the system’s propensity, or its readiness, for change; the journey or implementation process; and the external or outer context. For ease of access and readability, we have streamlined this model by rationalizing the number of variables that Greenhalgh et al. [12] stipulated in their original work. Of course, all models are simplifications of reality and even one that acknowledges a very large number of variables is, nevertheless, merely a model that reduces real-world complexity for the purposes of explication.

This is not to deny that there are, at the broadest levels, iterative roadmaps from bench to bedside or test tube to needle. However, this assessment does illuminate the reality that there are many components, moving parts, and shifting relationships, and that innovative journeys are much more convoluted, imprecise, uncertain, ambiguous, and deceptive than the pipeline proponents realized or hoped for. Social science had been waiting in the wings, eager to point this out, and have the mechanistic pipeline view excised. It brings to mind the English poet, David Whyte, who aphoristically said, “*Stop trying to change reality by attempting to eliminate complexity*” [17], and Abdus Salam, the Pakistani theoretical physicist and Nobel Prize winner, who once remarked, “*From time immemorial, man has desired to comprehend the complexity of nature in terms of as few elementary concepts as possible*” [18]. Yet, more mechanistic, simplified views of the world cannot wish away its complexity.

That said, there are some today who still persistently hold a traditional pipeline view, even in the face of experience with its shortcomings. At bedrock, this most likely has something to do with the architecture of the human mind, which often sees things in cause-and-effect terms [11, 19]. The brain has evolved to compose a narrative, linear account of events that unfold with a past–present–future representation of how things work [11, 19]; this forms part of the executive function of the brain responsible for planning, organizing, and reasoning [20]. Of course, the mind is also capable of out-of-the-box creative thinking, but straight-line rationalizing frequently trumps other ways of imagining how the world works.

## Complex Adaptive Systems (CAS) theory – raising the bar in the challenge to linearity

When we talk about the world being more complex than we typically imagine it to be, we do not just mean that it is complicated, or layered, or socially dense, or sometimes confusing. We do not mean, either, that it is merely unpredictable and varied, although it is certainly all of these things. We are also heralding the science of complex systems, which has developed, in part, out of systems theory, as a multi-disciplinary take on understanding many facets of the world (see Glossary of terms; Table 2).

**Table 2:** Glossary of terms

Glossary of terms	
Adaptation	The capacity to adjust to internal and external circumstances; usually thought of in terms of modifying behaviors over time
Agents	The individual components of a complex system – typically, individuals, whose capacity for sense-making means they can learn and adapt their behaviors across time, or artefacts
Complex Adaptive System	A dynamic, self-similar collectivity of interacting, adaptive agents and their artefacts
Complexity	The behavior embedded in highly composite systems or models of systems with large numbers of interacting components (e.g., agents, artefacts and groups); their ongoing, repeated interactions create local rules and rich, collective behaviors
Culture	The sum of the shared values, attitudes, and beliefs across part of or the whole of an organization (e.g., across the division of medicine, or an entire hospital or health service)
Emergence	Behaviors that are built from smaller or simpler entities, the characteristics or properties of which arise through the interactions of those smaller or simpler entities; the larger entities are one level up in scale, and manifest as social structures, patterns, or properties
Feedback loop	A recursive mechanism creating reciprocal behaviors that reverberate back in on themselves; a positive (self-reinforcing) feedback loop increases the rate of change of a factor, creating more of its own output; in a negative (self-correcting) feedback loop, the output responses dampen the change or modulate its direction
Implementation science	The processes of translating research into practice, understanding what influences translational outcomes, and evaluating the adoption of interventions
Network	An interlocking web of relationships or connections at varying levels of scale in a system; the agents or artefacts are the nodes and the relationships between them are lines or vectors, which together describe the structure of the interactions of the network’s membership
Path dependence	Current events and circumstances are influenced, and can be determined, by prior events and circumstances, harking back to the origins of the entity or system; path dependence underpins the point that ‘history matters’
Perturbation	An internal or external disruption or unexpected event that affects normal patterned behaviors, structures or processes; often thought of as an external disturbance or interruption to the current state-of-affairs
Self-organization	The way in which agents interact to coordinate their own circumstances, workplaces, processes and procedures, such that they order their work and they autonomously, or semi-autonomously, organize their localized behavior; this can occur passively or actively
Sensemaking	Methods by which individuals figure out what is going on around them; a typically social process among agents in which they come to a shared meaning of their experience, and is necessary for action in the face of ambiguity or uncertainty
Social network	A set of people who have relationships, communications, ties, or interactions that connect them
System dynamics	An analytical modelling methodology used for problem solving, which combines qualitative and quantitative data and identifies the fundamental elements of a system, and how they influence one another over time
Tipping point	A critical point in a system in which a kind of radical, potentially irreversible, change may occur, resulting in a different state of system behavior, which can settle into a new equilibrium

Complexity theory can be applied at multiple scales, from the very smallest, ranging from quantum foam to quarks, to the minutiae of the chemical and biological underpinnings of matter, to the behavior of molecules and cells, up to the macro interactions of humans, their groups, and even entire civilizations [21]. Complexity science has more recently been utilized in healthcare in order to apprehend, for example, the management, safety, and organization of clinical services [22, 23], as well as the implementation of interventions and the translation of evidence into practice [24].

Complexity science challenges conventional wisdom and an unduly straight-line approach to implementation on a number of fronts. Traditionally, people have studied parts of a system (the people, the intervention, the outcomes) as distinct variables, assuming the influences on one another to be straightforward [25], or at least knowable. These effects were conceived as additive, where the sum of the parts equaled the whole and a predictable relationship existed; that is, causes were identifiable because they preceded effects, and led to them. In designing interventions, people in this mode have aimed for reduction and control, removing the influence of, or controlling, ‘extraneous’ or ‘confounding’ variables [26]. Researchers and implementers then inferred the ability to generalize findings derived from this approach across contexts. Thus, an effect observed through well-controlled experimentation in one environment would be assumed to occur similarly in other situations; this may have worked in some cases, but by no means always.

In contradistinction, in complexity science, while the components of a system, namely the agents and their artefacts, are important, they are often secondary to the relationships between these components [27]. In such systems, agents communicate and learn from each other and from their environment, and adjust their behavior accordingly. However, there are many cross-cutting interconnections and influences. As such, the system is best described as a CAS, meaning that it has the capability to self-organize, accommodate to behaviors and events, learn from experience, and dynamically evolve [28], but not necessarily in ways anyone can forecast with any degree of confidence.

The self-organizing, iterative, reverberating interactions among agents, which in the healthcare CAS includes stakeholder groups such as doctors, allied health, patients, nurses, managers, and policymakers, as well as many other subgroups, give rise to unpredictability and nonlinearity, with causes and effects often disconnected or disproportional to one another [19, 25]. CASs are distributed in space and behave dynamically across time, with idiosyncratic interactions among agents at the local level determining the context, and the present and future behaviors of the system [24]. Through the interactions among the system’s components, global system patterns emerge and new factors (e.g., technology, policy, novel relationships, practices) eventuate.

These patterns are influenced by feedback loops, where different system inputs at different points in time perpetuate their own outputs, either dampening or enhancing them. Feedback helps to explain how responses to interventions, which might be positive at first, are often not sustained. The relatively loosely or tightly coupled interconnections between agents within a CAS, and their changeability over time, suggest there is much propensity for unintended consequences of an intervention in addition to the improvements agents hope for [29]. Borrowing from Gould and Eldridge’s famous distinction in evolutionary biology [30], health system progress in such circumstances resonates much more with the idea of punctuated equilibrium than that of morphologic gradualism.

## Enter implementation science

More recently, the efforts to study methods and mobilize knowledge, designed to enhance the ways in which we acquire and use evidence in healthcare, have been termed ‘implementation science’. For convenience, we can date this idea from the first issue of *Implementation Science* in 2006, although some scholars had been working on the development of this field before then. Implementation science is not a unified approach to getting evidence into practice, but rather comprises diverse perspectives, frameworks, and methods. However, broadly, implementation science is characterized by three aims, namely (1) to describe the process of translating research into practice (process models), (2) to understand what influences implementation outcomes (determinant frameworks, classic theories, implementation theories), and (3) to evaluate the implementation of interventions (evaluation frameworks) [31].

The two sciences of complexity and implementation need not be mutually exclusive, though they have been largely seen and treated as such. Nevertheless, some of what is published under the umbrella of implementation science is certainly antithetical to complexity science, drawing as it does from the linear, reductionist paradigms. Table 3 provides a brief comparison of the sciences of complexity and implementation, as well as how they might be fused.

**Table 3:** Comparison of some key characteristics of implementation science and complexity science and their integration

Features	Implementation science	Complexity science	Complexity science and implementation science
Task	The task is specific: getting evidence into clinical practice in an understandable way	The task is context dependent; properties of complexity apply to biology, ecology, physics, computer science, human social systems	Tailored solutions and iterative processes
Theoretical assumptions	Heterogeneous and diverse – numerous theories, frameworks, and models	Homogenous – core assumptions of complexity science are characterized by ‘universality’ (i.e., they apply across all complex systems)	Different theories, frameworks, and models require an understanding of complexity features such as unpredictability, uncertainty, emergence, interconnection
The intervention	To be standardized to permit generalizability	To be adapted to meet needs	Factoring in complex interventions and complex settings
The context	Full of confounders, a ‘problem’ to be solved for successful implementation	An intrinsic part of a complex system; a dynamic environment that must be factored in for any intervention to be successfully taken up	For improvement to be realized, the context must be re-etched or re-inscribed such that its culture, politics, and characteristics are altered
Historical underpinnings	Evidence-based practice movement, statistics, and the scientific method	Systems theory, chaos theory; emanating from diverse scientific disciplines	More sophisticated change models can be encouraged to arise over time
Aims within health services research	- Describing or guiding the process of translating research into practice (process models) - Understanding or explaining what influences implementation outcomes (determinant frameworks, classic theories, implementation theories) - Evaluating implementation (evaluation frameworks)	- Description of complex system • Understanding context • Relationships among agents • Dynamics • How rules and governance structures emerge, i.e., self-organization - For prediction rather than implementation	- Ensure that turning evidence into practice is accomplished without too many unintended negative consequences; improvement might be sustained, potentially through the adaptation of the intervention to different settings - Implementation is not merely based on effective planning but anticipation of a range of possible outcomes
Tools and methods	Randomized controlled trials, behavior change interventions, step-wedge designs	Causal loop diagrams, system dynamics modelling, network articulations	Realist evaluation, long-term case study, participatory research, stakeholder analysis, systems mapping, social network analysis

Sources: Authors’ conceptualizations and May et al. [24]; Braithwaite et al. [7]; Rapport et al. [65]; Hawe et al. [32]

Despite their differences, the two theoretical paradigms can be used together to the benefit of both theory building and healthcare practice and systems improvement. The complexity lens can help illuminate the scope of the implementation problem to be tackled and the dynamics of change and inertia. The translation of evidence into new clinical or organizational practices does not unfold in a static and controlled environment awaiting the attention of top-down change agents; it takes place in settings comprised of diverse actors with varying levels of interest, capacity, and time, interacting in ways that are culturally deeply sedimented, and have often solidified [32, 33]. In other words, the complex patterns by which healthcare is delivered, and the enmeshed social structures inherent within the system, are already established and entrenched. In such a networked, at times tightly and at others loosely coupled ecosystem, already teeming with activity and relationships, knowledge uptake is rarely simple or straightforward, and has to find a place in an intricate, pre-existing milieu.

Going further, spread is closely related to uptake. The patterns of interaction between agents and their environment are locally specific, and although they share features with other CASs, they also exhibit remarkable variation from one site to the next. The notion, then, that a new practice can be adopted equally well and in the same manner across a whole health system, is untenable. Thus, standardization of an intervention, and assuming its generalizability, can be the downfall of successful implementation [34].

However, implementation scientists, or at least those working within implementation science with pluralistic conceptualizations of the world, have not been standing still. The need to factor in context is being increasingly recognized by scholars in implementation science, as is the identification of barriers and facilitators to an intervention [35]. For example, the Promoting Action on Research Implementation in Health Services formula [36] sees successful implementation as a function of the explicit interrelations among evidence, context, and facilitation. Nevertheless, these contextual characteristics of the environment are often viewed as ‘confounders’ in implementation research, rather than the normal conditions of practice in healthcare. Complexity science, in highlighting the dynamic properties of every CAS and the local nature of each system’s culture, suggests that what operates as a ‘barrier’ to implementation in one site may not do so in another, and could even be facilitative [24].

## Informing implementation with complexity

In complexity-informed approaches to implementation it is not enough to leverage facilitators or eliminate barriers; the focus of implementation shifts from the fidelity of the intervention to its effective adaptation [37, 38]. Thus, Hawe et al. [34] argue that, rather than standardizing aspects of an intervention, despite some essential functions being replicable, the form of an intervention should be varied as required by context [39]. This type of CAS-oriented approach is particularly important when attempting to scale-up or spread interventions previously found to be effective in one, or a limited number of sites, to the whole system. Improvement structures may thus involve tailoring to context and harnessing the self-organizing and sense-making capacities of local agents [38]. Indeed, working with bottom-up local stakeholders is paramount to adapting an intervention to their practices, facilitating ways to get them onboard with the intervention, in piloting it, in reflecting on progress amongst stakeholders, and in providing feedback to participants to help them embrace implementation iteratively over time. In such a messy, complex set of circumstances, it makes less and less sense to think of ‘knowledge producers’ as conceptually distinct from ‘knowledge users’ [40] when indeed they are inter-related.

Chambers et al. [41] suggest that a further consideration is the sustainability of an intervention. Sustainable change requires the ongoing adaptation of an intervention to multilevel contexts, with expectations for lasting improvement rather than diminishing outcomes over time. In this regard, implementation in the hands of complexity theorists is increasingly recognized as an iterative and recursive, long-term process rather than a linear one [35]. Complexity science thereby encourages not only attention to the context of an intervention, but also to the interactions between elements and the consequences of this intervention for the system. The implementation method of choice will not necessarily be the randomized clinical trial or experimental design, but will be the iterative and responsive, more ecology-aware, social science-informed approaches such as those envisaged by longer term realist designs or process evaluation of implementation efforts [32, 42].

**Fig. 3:**
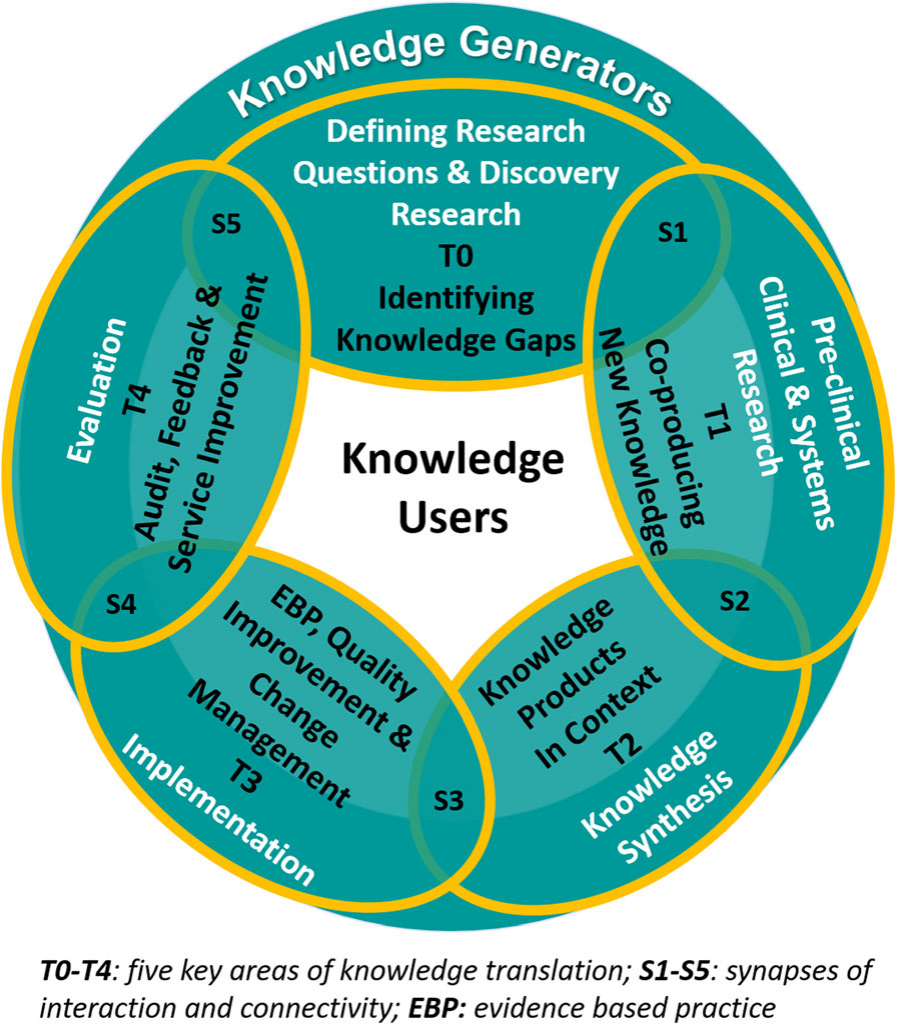
Process of developing a model of knowledge translation aligned to complexity science. Source: Modified from Kitson et al. [40]. Use of this image is supported by a Creative Commons License http://creativecommons.org/licenses/by/4.0/

Despite the potential utility in harnessing complexity science for implementation, until now, not much conjoining of the two, either theoretically or empirically, has occurred. There have been intermittent examples of using a complex systems framework to inform clinical transformation, as when Best et al. [43] applied complexity thinking in the implementation of new clinical guidelines in British Columbia, Canada. They noted that the implementation of the guidelines required the ability to tailor system-level recommendations to local context. In another promising turn, there have been more recent attempts to explicitly challenge the pipeline view of knowledge translation, with Kitson et al. [40] undergoing an iterative process to develop a complexity-informed model that highlighted the connections between phases previously conceptualized as discrete such as problem identification and knowledge synthesis. This model (Fig. 3) in essence highlights the key issues to be considered, including the distinctions and connections between knowledge users and knowledge generators, the importance of arriving at good definitions for the gaps, and co-producing new knowledge and contextualizing it, as well as implementation and evaluation.

That said, a recent systematic review by Brainard et al. [29] found that health interventions using complexity science approaches have done so inconsistently, for example, often not incorporating an evaluation component or failing to analyze the potential, unintended consequences of the intervention. Nevertheless, this recent work has suggested the value of complexity science in creating large-scale system transformation, including sensitizing stakeholders to the natural properties of CAS that might then be leveraged by emphasizing distributed leadership, networks, sense-making, and feedback loops [38, 42, 44].

Thus, thinking is altering, at least amongst some leading theorists and researchers, and we are now more advanced in understanding systems change, with new models replacing the pipeline approach. Having established the juxtaposition of complexity and implementation, we now examine how some of these ideas have been leveraged to accomplish large-scale system transformations in Australia, exploiting the combined complexity–implementation paradigm.

### Case 1: Rapid response systems and the New South Wales ‘Between the Flags’ (BtF) program

Since the 1980s, there has been an increasing focus on patient safety and quality of care in hospitals internationally, as well as in Australia. Many initiatives have been designed and conducted, but there is limited evidence to show that systems-level improvement has been achieved [45]. One notable exception has been the implementation of rapid response systems (RRSs), in which specialized teams attend to inpatients whose deteriorating condition has been identified through reference to a set of defined criteria. RRSs have had a significant impact on patient safety, with evidence that they have reduced inpatient mortality and cardiac arrests by about one-third [46, 47]. Yet, RRSs illustrate that even a relatively simple and intuitively sound intervention can struggle to be adopted into the CAS of healthcare, where history, path-dependence, and context, especially social influences, can have substantial effects.

RRSs were a bottom-up initiative, coming from self-organizing clinicians who recognized that the deterioration of a patient’s condition could easily go undetected until it was too late to reverse. In their chapter outlining the history of the RRS in Australia, Braithwaite et al. [48] described the strong influence of context on the adoption of this intervention. Attempts in the early 1980s to introduce a Medical Emergency Team (MET), the precursor of RRSs, failed in a large London teaching hospital due to inertia and unconcealed opposition, but succeeded in a smaller, more recently established teaching hospital in Liverpool, New South Wales (NSW), Australia. Barriers and confounders of the London adoption were identified as the entrenched medical and management hierarchies, and an onerous bureaucracy. Perhaps more significantly, there were strongly deterministic path dependencies, represented by a pervasive belief in medical culture that patients were ‘owned’ by their admitting doctor, a belief that clouded who was authorized to treat and where accountability for patients lay. In Liverpool, innovation was more accepted, medical autonomy less jealously guarded, and there was a culture of readiness for experimentation and change.

The notion of the MET began to be taken up in other countries without active implementation mechanisms. Through deceptively simple knowledge dissemination means, such as articles in low-impact publications or conference presentations, and clinical networks and informal discussions, clinicians assessed their needs and adopted METs, tentatively at first, into their own context [49]. This highlights that, while an implementation plan is typically necessary for system-wide change, bottom-up, knowledge dissemination approaches can facilitate attitude change. That is, interconnected clinicians communicate locally and across the boundaries of their systems, influencing one another in their own and other environments, and self-organizing their practices in novel ways based on this new knowledge. This type of on-the-ground interactivity, whereby clinicians felt ownership of the incremental changes rather than having it imposed on them, made possible the eventual system-wide transformation.

The tipping point for dissemination of many large-scale, system-wide changes has been in the form of a perturbation to the system, such as the SARS epidemic in Canada or the tragic death of teenager Vanessa Anderson in NSW, Australia [50]. This latter case, deemed a preventable death caused by failure to recognize the teenager’s deteriorating condition, led to the BtF program, which flipped the bottom-up approach of previous MET implementations into a whole-of-system approach with concerted support from multiple sectors, including government [51].

BtF alludes to the Australian Surf Life Saving model that offers surveillance of bathers on popular surf beaches, who swim between two yellow and red flags, planted conspicuously in the sand. Surf Life Saving Australia estimate that they rescue 35 swimmers under threat of drowning and intervene in 913 other cases per hour on a typical summer’s day using this simple model. The BtF program used the imagery of a safe zone to redesign and standardize vital sign charts across the hospital system [52], with upper and lower unsafe limits reflecting the colors of the flags (yellow as early deterioration warning sign, red as late). Vital sign readings that were in the yellow zones triggered an urgent clinical review and the red triggered intervention by the specialized MET. The work was led by the Clinical Excellence Commission, an agency set up to oversee quality and safety across NSW healthcare.

For a linear thinker, this highly effective intervention would seem easy to implement with predictable, positive outcomes. However, the issue is not the relative simplicity of the model of monitoring a patient’s vital signs with a standardized form and the use of a MET intervention to ‘rescue’ them when straying into the unsafe yellow or red zones, but rather the complexity of the system into which the intervention is being introduced. BtF was implemented into NSW’s 225 public hospitals in January 2010. Many had already adopted RRS-style models in idiosyncratic ways. For its successful introduction, the Clinical Excellence Commission recognized the complexity of the system, including the independence and interdependence of agents, the presence of positive and negative social influences, and the generation of possible adverse knock-on effects. Accordingly, the program had five elements, namely governance, standard calling criteria (the red and yellow flags), a two-tiered RRS in each facility, an associated education program, and an evaluation plan. Governance mechanisms supported by well-staffed and supportive advisory boards, alongside a State-wide policy directive, held hospitals to an implementation schedule with scope for local flexibility and promulgated clearly defined roles and expectations. The standard calling criteria were incorporated into the new, mandatory NSW standard observation charts with a simple track-and-trigger design.

The two-tiered RRS response was developed to prevent the problem of false positives that could overwhelm the system, as well as false negatives that would result in failure to rescue [53]. Both types of errors could undermine the credibility of the program and lead to poor clinical compliance on the wards. BtF designers also understood the challenge of embedded social influences such as medical hierarchies and clinical tribalism [48]. The program diffused authority for intervention from medical consultants to any health professional detecting a patient outside the flags.

Following the extensive preparation period, uptake was rapid. Clinician fears of ‘extra paperwork’ were shown to be unfounded and the empowerment of nursing and junior medical staff to initiate a rescue reinforced its utility. Evaluation data, as it was collected, showed consistent falls in cardiac arrest and mortality rates (cardiac arrest by 42%; *P* < 0.05) and the rapid response rate increased by 135.9% (*P* < 0.05) [53].

Thus, BtF showed that successful implementation requires an understanding of the complex system into which even ‘simple’ interventions are being introduced. CAS theory can help to unpack the multi-dimensional contextual issues and address them with multifaceted solutions prior to the roll out of such a large-scale intervention.

### Case 2: New nation-wide safety and quality standards

In 2013, systems-level reform of the Australian accreditation model occurred with the implementation of the Australian Health Service Safety and Quality Accreditation Scheme. A critical component of the scheme, overseen by the Australian Commission on Safety and Quality in Health Care (ACSQHC), has been the development and application of new National Safety and Quality Health Service Standards (NSQHSS). The development of the 10 standards represented an important element in the safety and quality of care architecture of the health system. The standards cover areas including governance arrangements, partnerships with consumers, and eight key clinical areas of health service operation (Box 1).

Each standard has a set of criteria, and for each criterion, a series of actions are required to be fulfilled. To achieve accreditation status, all core actions for health services must be demonstrated. The work has drawn international interest and is informing efforts to improve the safety and quality of healthcare in other countries [54].

The Australian Health Service Safety and Quality Accreditation Scheme has been enacted with an appreciation of the CAS features of healthcare, and the implementation process was dynamically modified in response to the multifarious and interlinked institutions, groups, and structural arrangements that can hinder or facilitate implementation, and must ultimately adopt the model. International experience shows that the inherent complexity of healthcare and in-built resistance, regardless of country, can be an impediment to adoption of such systems-level reforms [55–58].

To respond to this challenging environment, the ACSQHC undertook extensive consultation activities with the aim of determining appropriate methods of utilizing existing government legislative powers to support the reform measures, to align the views and actions of diverse groups, and to foster distributed leadership across reform elements [59–61]. In total, the ACSQHC arranged 227 separate consultation activities involving over 1000 stakeholders spanning the breadth of the Australian health system. The perceived importance of these activities for maximizing the effectiveness of the scheme reinforces the fundamental role of continued stakeholder engagement as a necessary facilitator of national reform [54]. The need for effective stakeholder engagement has also been identified in relation to other systems-level healthcare reforms internationally [62, 63]. The ACSQHC continues to undertake consultation with health services to facilitate effective implementation of the scheme and further revisions have been made to the standards over time (in 2016 and again in 2017), assuring their continued relevance [59–61].

Despite the nature of the standards’ implementation as seemingly a top-down, government-sponsored, homogeneous model, NSQHSS have been well received by the system due to the clinical focus of most of the standards. This was considered crucial for increasing the engagement of health professionals and board members in health and quality improvement activities [54]. Participants proposed that the NSQHSS provided, for the first time, a clearly evidenced-oriented, coherent, and integrated national framework. The scheme separated and clarified responsibilities of different actors for accreditation standards development, surveying processes and decisions, and regulation and policy matters. As a result, the initiative has been seen to mobilize expectations, integrate roles and responsibilities, and promote transparency [54].

From the outset, two potential risks to the credibility of and satisfaction with the scheme at the health system level were raised, namely the application of the NSQHSS across varied settings and the reliability of assessments by different accrediting agencies. The application of the NSQHSS across settings was discussed in the consultations as a point of credibility – that the same expectations would be applied to different health services, in different settings, was considered vital to the government’s interests in equity [54].

**Fig. 4:**
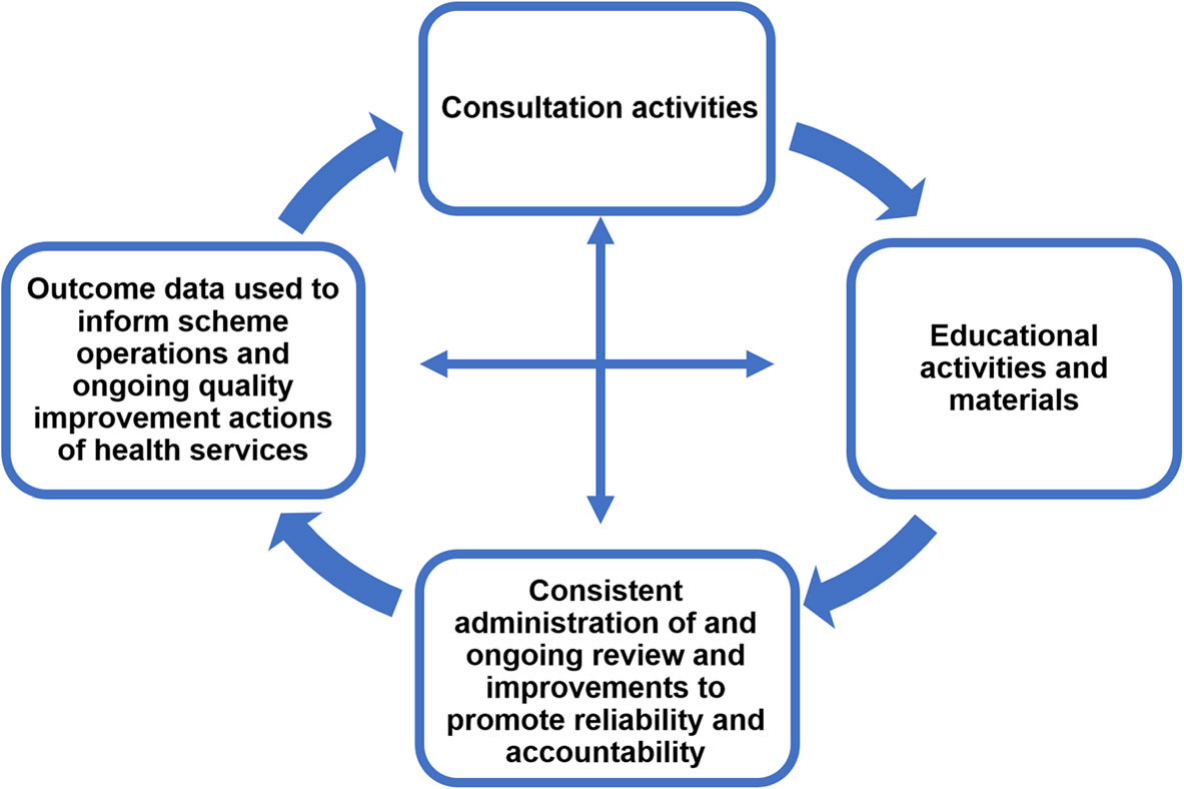
Strategies facilitating implementation. Source: Greenfield et al. [54] Permission granted by John Wiley and Sons for use of this image. License number: 4236860320684

Four strategies to facilitate implementation, to reinforce the potential benefits, and to overcome the substantial challenges facing the scheme emerged (Fig. 4). The widespread ACSQHC consultation activities were seen to facilitate implementation by providing a common platform for knowledge transfer, encouraging widespread stakeholder engagement. At those meetings, high-quality, accessible educational activities and materials were provided. Feedback loops in the form of regular review of the program and updates to the system using progress data helped maintain momentum.

## Discussion

Pipeline models sprang initially from those adhering to a linear worldview of the path from knowledge creation, through knowledge products, to knowledge use. The task was to get evidence into practice, and this was seen by many as a simple, staged activity, following recipe-style models such as the one expressed by Cooksey [3]. In the minds of many scholars and practitioners, including some who self-define as implementation scientists, the process of bench to bedside has, by and large, continued to be conceptualized in a largely mechanical frame, although some researchers and theorists have introduced complexity ideas to it [7, 40, 64]. Complexity science offers a radically different set of considerations to those interested in systems change. As a paradigm, it denies over-simplification, and is conceptually transformative, adding a much richer set of understandings to the task of systems improvement.

The two traditions of implementation science and complexity science can be drawn together, and culminate in more textured, multi-dimensional, complexity-informed models. Paradigm-shifting exemplars that have achieved this include those offered by Greenhalgh et al. [12] on innovation (Fig. 2) and Kitson et al. [40] on knowledge transfer (Fig. 3).

The RRS case was bottom-up followed by top-down; the accreditation case was top-down but with middle-out and bottom-up responses. Whether top-down, middle-out, or bottom-up, these Australian case exemplars show how complexity science attributes (emerging ideas, iterative approaches, feedback mechanisms, inter-dependencies, building momentum over time, dynamic communication with multiple stakeholders, systems perturbation) can be factored into change programs. Both cases involved extensive coalition building over multiple years in order to reach a tipping point. We provide a synthesis of what we have learned from this theoretical analysis of implementation science and complexity science by using the case exemplars to empirically illuminate the interface of the two paradigms (Table 4). These case studies show that successful systems change can take varied forms and that the implementation sequence can differ depending on circumstance and needs. Thus, a hybrid of factors drawn from implementation science and complexity science help explain how systems change occurred in these two case exemplars.

**Table 4:** Case study comparisons – exemplifications of implementation science and complexity science paradigms

Selected implementation or complexity characteristic	Case 1: Rapid response systems’ adoption and spread	Case 2: Introduction of national quality standards
Overarching strategy and implementation sequence	Bottom-up followed by top-down implementation, with middle-out support	Top-down with localized middle-out and then bottom-up acceptance
Adaptation	Localized arrangements, then accommodating to an agreed, state-wide model	Legislated authority; brokered national agreement following extensive consultations
Agents	Clinicians in intensive care units; later, managers and policymakers; acceptance by admitting clinicians in wards	Policymakers and regulators; accreditation agencies; organizational adoption
Culture	Positive values and attitudes amongst intensivists; eventual behavior and practice change across the system	Policy enactment from the highest levels as a driver of eventual change through the hierarchy
Feedback	Local clinicians influencing each other recursively for many years; eventually, formal design and implementation to reinforce and institutionalize the agreed framework	Policy implementation model: Ministerial endorsement, ongoing consultation and education leading to dampening of opposition and widespread take-up and adoption
Networks	Intensive care physicians as prime movers; later, policymakers, managers, and other clinicians	Policy and accreditation bodies, with research partners lending expertise and support
Path dependence	Thirty years in the making, leading to eventual acceptance against systems and clinical inertia	Ten years of policy and managerial discussion and maneuvering before implementation
Type of perturbation	Gradual radiation of acceptance over time nationally and internationally	Legislation as an enabler, acting as an initial mover
Self-organization	Intensive care physicians particularly; followed by whole-of-system acceptance	Influence groups of policymakers, managers and academics followed by big-bang introduction
Tipping point	Growing acceptance by clinicians leading to leaders eventually invoking the authority of the Clinical Excellence Commission	Ministerial authority, legislative enactment, sustained pressure from peak bodies, eventual system-wide acceptance

The key is to harness such understanding to strengthen progress with other multifaceted health systems interventions. Based on these examples, the portents are for future change agents to conjoin complexity science and implementation science approaches for the benefit of systems-level change.

## Conclusion

Notwithstanding this analysis and these case exemplars, we conclude with a word of warning. Complexity thinking adds a real-world, multidimensional appreciation of the system and its density and dynamics, but it does not make it easier to effect change; in fact, the opposite is true. We can no longer assume to solve health systems issues by pretending or conspiring to imagine that they have Newtonian properties, and pipeline models should be seen for what they always were – idealistic, normative renderings of the world. Even though this makes our ambitions to improve healthcare infuriatingly more difficult, we must grapple with the world we actually inhabit, not the one we wish we did.

## Box 1: The 10 National Safety and Quality Health Service Standards

1. Governance for safety and quality in health service organizations

2. Partnering with consumers

3. Preventing and controlling healthcare associated infections

4. Medication safety

5. Patient identification and procedure matching

6. Clinical handover

7. Blood and blood products

8. Preventing and managing pressure injuries

9. Recognizing and responding to clinical deterioration in acute healthcare

10. Preventing falls and harm from falls

Source: Australian Commission on Safety and Quality in Health Care [59].

## Abbreviations

ACSQHC: Australian Commission on Safety and Quality in Health Care
CAS: Complex Adaptive System
MET: Medical Emergency Team
NSQHSS: National Safety and Quality Health Service Standards
NSW: New South Wales
RRS: Rapid Response System
